# Health research methodology education in Canadian emergency medicine residency programs: A national environmental scan

**DOI:** 10.36834/cmej.68753

**Published:** 2020-09-23

**Authors:** Aaron Wang, Allison Meiwald, Robert Harper, Kristine Van Aarsen, Justin W. Yan

**Affiliations:** 1 Schulich School of Medicine and Dentistry, Western University, Ontario, Canada; 2 The Division of Emergency Medicine, Department of Medicine, London Health Sciences Centre, Ontario, Canada; 3 University of Cincinnati College of Medicine, Division of General and Community Pediatrics, Cincinnati Children’s Hospital Medical Center, Ohio, USA

## Abstract

**Objectives:**

Our objective was to describe the variability of research methodology teaching among English-speaking Royal College of Physicians and Surgeons of Canada emergency medicine (RCPSC-EM) residency programs. We also aimed to identify barriers to teaching research methodology curricula.

**Methods:**

An electronic survey was sent by email to program directors and residents of English-speaking RCPSC-EM training programs countrywide. Reminder emails were sent after two, four, and eight weeks. Quantitative, descriptive statistics were prepared, and qualitative data and themes were identified.

**Results:**

We received a total of seven responses from the possible 12 program directors (response rate = 58.3%). Out of 354 potential resident respondents, 82 (23.2%) completed the survey. There was disparity between resident and program director responses with respect to the existence of curricula, preparation for Royal College exams, and usefulness for future practice. Barriers to teaching a research methodologies curriculum included lack of time, support, educated faculty, and finances.

**Conclusion:**

This survey demonstrates that Canadian EM residency programs vary with respect to research methodology curriculum, and discrepancies exist between residents’ and program directors’ perceptions of the curriculum. Given the lack of a standardized research methodology curriculum for these programs, there is an opportunity to improve training in research methodology.

## Abstract (fr)

**Objectifs:**

Notre objectif vise à décrire la variabilité de l’enseignement des méthodologies de la recherche entre les programmes anglophones de résidence en médecine d’urgence du Collège royal des médecins et chirurgiens du Canada (MU - CRMCC). Nous avions également pour but de déterminer les obstacles qui entravent l’enseignement des cursus de méthodologie de la recherche.

**Méthodes:**

On a transmis par courriel un sondage électronique aux directeurs de programme et résidents des programmes anglophones de formation MU - CRMCC dans tout le pays. Des courriels de rappel ont été envoyés après deux, quatre et huit semaines. On a ensuite préparé des statistiques descriptives quantitatives et identifié des données et thèmes qualitatifs.

**Résultats:**

Nous avons reçu un total de sept réponses des 12 directeurs de programme actuels (taux de réponse = 58,3 %). Quatre-vingt-deux des 354 résidents potentiels, soit 23,2 %, ont répondu au sondage. Nous avons relevé une disparité entre les réponses des résidents et celles des directeurs de programme en ce qui a trait à l’existence du cursus, la préparation en vue des examens du Collège royal et l’utilité dans la pratique future. Parmi les obstacles entravant l’enseignement des cursus de méthodologie de la recherche, on retrouvait le manque de temps, de soutien, de corps professoral qualifié et de fonds.

**Conclusion:**

Ce sondage démontre que les cursus de méthodologie de la recherche des programmes de résidence en médecine d’urgence varient dans l’ensemble du Canada et que des divergences existent entre le point de vue des résidents et celui des directeurs de programme sur ces cursus. Étant donné l’absence d’un cursus uniforme de méthodologie de la recherche pour ces programmes, ceci nous offre une occasion d’améliorer la formation en méthodologie de la recherche.

## Introduction

Since the advent of emergency medicine (EM), researchers have argued the importance of incorporating research education into EM residencies, as it benefits both the academic community and learners.^1^^,^^2^ With the implementation of Competency Based Medical Education (CBME) by EM residency programs in Canada^3^, there are three research-related Entrustable Professional Activities (EPAs) residents are expected to meet. These EPAs are: appraising and integrating new evidence into clinical practice; advancing emergency medicine through a scholarly project; and participating in a quality improvement initiative to enhance patient care.^4^ The Royal College of Physicians and Surgeons of Canada (RCPSC) also identifies the value of understanding the scientific principles of research for all physicians.^5^ Despite these objectives and specific EPAs, we do not know the health research methodology curriculum (i.e. the education provided to residents to help them develop ideas and perform research) and scholarly project expectations at the 12 English-speaking RCPSC-EM training programs in Canada.

We hypothesize that since there is no explicit, standardized health research methodology curriculum, the health research methodology curricula across the 12 English-speaking EM training programs in Canada is variable, with respect to content, delivery, and requirements. This variability may identify the need for a standardized national curriculum, as well as potentially identify domains where programs may improve. The purpose of this study is to explore this variability by determining the amount and perceived usefulness of health research methodology curricula that exists in English-speaking EM residency programs. Our secondary objective was to identify and describe any barriers to teaching health research methodology curricula.

## Methods

### Study population

All RCPSC-EM program directors and residents in English-speaking programs were surveyed regarding research methodology curricula. Program directors and residents at French-language residencies were excluded due to language barriers. The study was approved by the Health Sciences Research Ethics Board at Western University.

### Survey development

Surveys for both RCPSC-EM program directors and residents were created using SurveyMonkey™ (see Appendix 1 and 2). Survey domains were developed after thorough consultation with local CBME and research leads. The survey development process included a thorough literature search, defining key terms such as research methodology, and optimizing the wording of questions for easier comprehension. Quantitative portions of the survey asked whether a curriculum exists as part of their residency program. 7-point Likert rating scales were used to assess the usefulness, appropriateness, ease of understanding, and applicability of the curriculum. The qualitative portion asked for a description of what currently exists and an explanation of the reasons for the quantitative answers (eg. why they felt a new curriculum was necessary). Residents were asked about their thoughts on the adequacy of their local curriculum, as well as potential barriers to teaching research methodology curriculum.

### Survey administration

Potential participants received via email an information letter approved by the local Research Ethics Board along with the link to the survey created by SurveyMonkey™. It was indicated in this information letter that completion of the survey implied consent. The survey was sent to RCPSC-EM program directors through email from September 24-November 26, 2018. Program directors' contact information was publicly available on the Canadian Residency Matching Service website.^6^ Program administrators were asked to distribute the invitation to residents, and reminder emails were sent to program directors at the 2-, 4-, and 8-week marks. Participation for both surveys was voluntary, and no monetary incentives were provided.

### Analysis

Quantitative, descriptive statistics were prepared using Microsoft Excel 2016 (Version 1910) and qualitative data were explored. Due to the descriptive nature of the study, statistical significance testing was not performed. Proportions of respondents were reported.

## Results

Of a possible 12 program directors, seven completed the survey (58.3%), with the majority being from institutions in Ontario and western provinces. Compared to sites where program directors did not respond, these institutions were larger with respect to the average number of trainees. All 12 program directors distributed the survey to their resident groups. Out of 354 potential resident respondents, 82 (23.2%) completed the survey.

When questioned about their research methodology curriculum, 58.5% of residents indicated a curriculum existed at their university. This contrasts with 100% of programs directors responding that such a curriculum exists. Most of this curriculum was offered in small or large group settings. Frequency was variable, occurring less than yearly (18.5%), yearly (20.4%), twice a year (9.3%) and more than twice a year (14.8%). Many residents (52.4%) and program directors (85.7%) said the curriculum was mandatory. Research staff and staff physicians were responsible for providing the curriculum. Only 25.6% of residents felt they were assessed or tested on the information provided. Just over half (57.1%) of program directors responded that the residents were assessed or tested on the information. Less than half of residents (34.2%) and most program directors (85.2%) felt the research methodology curriculum prepared them for their Royal College exams. Only 39.0% of residents felt it prepared them for their future as a consultant physician while all (100%) program directors believed it did. When asked to comment on barriers to a research methodologies curriculum, program directors indicated that lack of time, educated faculty, and finances were contributing factors. Of all resident respondents 83.7% said they would use or consider using a web-based curriculum module regarding research methodology if available. Full results from surveyed residents can be found in Table 1.

**Table 1:** Research methodology curriculum responses

	All residents (*n* = 82)*	Residents where PD responded (*n* = 54)		Program Directors (*n* = 7)
Does a research methodology curriculum exist?				
Yes No Unsure Unanswered	48 (58.5%) 6 (7.3%) 14 (17.1%) 14 (17.1%)	35 (64.8%) 3 (5.6%) 8 (14.8%) 8 (14.8%)		7 (100%) 0 0 0
Is attendance mandatory?				
Yes No Unsure Unanswered	43 (52.4%) 1 (1.2%) 1 (1.2%) 37 (45.1%)	33 (61.1%) 1 (1.9%) 0 20 (37.0%)		6 (85.7%) 1 (14.3%) 0 0
Are residents assessed on material?				
Yes No Unsure Unanswered	21 (25.6%) 10 (12.2%) 14 (17.1%) 37 (45.1%)	20 (37%) 7 (13%) 7 (13%) 20 (37.0%)		4 (57.1%) 3 (42.9%) 0 0
Does the curriculum prepare residents for the Royal College exam?				
Strongly Disagree Disagree Neither agree/disagree Agree Strongly Agree Unanswered	2 (2.4%) 1 (1.2%) 14 (17.1%) 20 (24.4%) 8 (9.8%) 37 (45.1%)	0 0 10 (18.5%) 16 (29.6%) 8 (14.8%) 20 (37.0%)	0 0 1 (14.3%) 4 (57.1%) 2 (28.6%) 0	
Does the curriculum prepare residents for future practice?				
Strongly Disagree Disagree Neither agree/disagree Agree Strongly Agree Unanswered	3 (3.7%) 1 (1.2%) 9 (11.0%) 21 (25.6%) 11 (13.4%) 37 (45.1%)	1 (1.9%) 1 (1.9%) 6 (11.1%) 16 (29.6%) 11 (20.4%) 19 (35.2%)	0 0 0 4 (66.7%) 2 (33.3%) 1 (14.3%)	

## Discussion

This environmental scan regarding health research methodology teaching at Canadian

RCPSC-EM residency programs has shown that residents have the perception that a health research methodology curriculum does not exist at all English-speaking programs; this curriculum seems to be inconsistent in the programs where it does exist according to discrepancies in residents’ and program directors’ responses. This has important implications, as there are national standards regarding research education, as set out by the Royal College, for EM specialist physicians completing residency.

A strong research methodology curriculum should have mentors that add to a supportive research environment, along with protected time for training in basic research methods.^7^ Our resident respondents may be wanting in this, as our study showed that residents feel a lack of time, support, educated faculty, and finances were barriers at their university. A U.S. study that surveyed EM residents who had completed their in-training exam showed that though residents were confident overall in their programs, they did not have positive opinions regarding their training in specific academic skills that could help in their future academic careers.^8^ These include skills like study design or statistical analysis. In our study, similarly, only 39% of residents believed their research methodology curriculum adequately prepared them for their future career as consultant physicians. However, knowledge of research methodology is encouraged for all consultant physicians in their role as a life-long scholar and ultimately for improved health outcomes for patients.^5^^,^^9^

When comparing the level of training in future physicians, similarities are seen with regards to interest in research. It was shown that Canadian medical students at Queen’s, Ottawa, and Western Universities were surveyed about their attitudes toward research in medical school.^10^ 43% were not actively involved in research and felt that barriers included time, lack of mentors, lack of formal teaching with respect to research methodology. Likewise, the findings of our study are comparable and suggests that barriers exist at multiple levels of medical education.

In our study, several responses differed greatly between residents and program directors. One difference was noted when participants were asked about the actual existence of a research methodology curriculum. While the majority (58.5%) of residents indicated a formal curriculum existed, 100% of program directors responded that a curriculum was in place. Interestingly, less than half (39.0%) of residents felt the research methodology curriculum prepared them for their future as a consultant, while 100% of program directors did. We speculate that residents may be unaware of what is included in the research methodology curriculum at their program. If residents are unaware of what is being offered or what is testable on exams, they may be unsure if it is useful to their career.

With Royal College EM having moved to a CBME assessment strategy nationally as of July 2018, many questions remain regarding the EPAs surrounding health research methodology education. Possible recommendations on how to improve the capacity of residency programs to meet these EPAs include providing standardized, accessible learning material that emphasizes the value of research methodology. Current literature suggests that using adaptive online modules to supplement learning on weaker subject areas as determined by pretests can potentially reduce the amount of time spent learning and can also increase residents’ satisfaction.^11^^,^^12^ This potential for extra learning time also addresses the barrier of not enough time to learn everything in a research methodology curriculum as noted by program directors in our survey.

## Limitations

We recognize several limitations in this study. This was a cross-sectional survey of English-speaking RCPSC-EM program directors and residents, which was subject to self-reporting and recall biases. As participation was voluntary, there may also be response bias; program directors have a good understanding of the overall curriculum of their program but may not have specific knowledge of the objectives relating to health research methodology. As well, they may be pressured to answer questions in a way that favorably reflects their programs. For the survey development process, we were unable to pilot test the survey, which could lead to reduced participant feedback with regards to survey length, question clarity, and relevance. The response rate of 23.2% for residents and 58.3% for program directors for this environmental scan is quite low. Since just over half of the program directors responded, in order to compare the resident and PD responses, we limited the analysis to responses from residents from programs where the program director also responded. This has the potential to provide an incomplete statistical and qualitative analysis.

### Next steps

Our study has shown that health research methodology curricula is variable and in fact does not even exist at some EM residency programs. Where it does exist, there are questions regarding its utility. Future projects should focus on the development of a national online curriculum that all residents have access to, that is evidence-based, and can be studied to examine its effectiveness.

### Conclusion

Health research methodology training within EM residency programs has been recognized as an important area of education for training competent EM physicians. This cross-sectional survey reveals variability in the health research methodology curricula in English-speaking RCPSC-EM residency programs.
